# Inadequate receipt of ANC components and associated factors among pregnant women in Northwest Ethiopia, 2020–2021: a community-based cross-sectional study

**DOI:** 10.1186/s12978-023-01612-0

**Published:** 2023-05-04

**Authors:** Abebaw Addis Gelagay, Tadele Biresaw Belachew, Desale Bihonegn Asmamaw, Desalegn Anmut Bitew, Elsa Awoke Fentie, Abebaw Gebeyehu Worku, Debrework Tesgera Bashah, Nigusie Birhan Tebeje, Mignote Hailu Gebrie, Hedija Yenus Yeshita, Endeshaw Adimasu Cherkose, Birhanu Abera Ayana, Ayenew Molla Lakew, Wubshet Debebe Negash

**Affiliations:** 1 Department of Reproductive Health, Institute of Public Health, College of Medicine and Health Sciences, University of Gondar, Gondar, Ethiopia; 2 Department of Health Systems and Policy, Institute of Public Health, College of Medicine and Health Sciences, University of Gondar, P.O. Box: 196, Gondar, Ethiopia; 3 School of Nursing, College of Medicine and Health Sciences, University of Gondar, Gondar, Ethiopia; 4 School of Midwifery, College of Medicine and Health Sciences, University of Gondar, Gondar, Ethiopia; 5 Department of Obstetrics and Gynecology, Zewuditu Memorial Hospital, Addis Ababa, Ethiopia; 6 Department of Epidemiology and Biostatistics, Institute of Public Health, College of Medicine and Health Sciences, University of Gondar, Gondar, Ethiopia

**Keywords:** Antenatal care, Inadequate ANC, Dabat, Ethiopia

## Abstract

**Background:**

Women's health and pregnancy outcomes are directly depends on the extent of ANC components received during their ANC visits. There are limited information about the components of ANC and associated factors. Therefore, the aim of this study was to assess the magnitude of inadequate recipient of ANC components and associated factors in northwest Ethiopia.

**Methods:**

This is a community based cross sectional survey conducted in Dabat Demographic and health survey from December 10/2020 to January 10/2021 among women who gave birth within two years before the survey. This study applied a census method to identify and select eligible pregnant women. A structured and pretested questionnaire was used to collect the data. The data was entered into Epi-data version 3.1 and exported to STATA version 14 for analysis purpose. Adjusted Odds Ratio at 95% confidence interval was used to show the association between dependent and independent variables. Statistical significance was declared at a *P* value less than 0.05.

**Results:**

A total of 871 pregnant women were identified from the survey and included in this study. Overall, 96.67% (95% CI: 95.24, 97.67) had not get adequate (all components) ANC. The components of ANC services were increased from 3.35 to 32.34%, 2.52 to 46.33% 1.96 to 55.8%, 2.31 to 46.53%, 3.54 to 55.75%, 2.46 to 44.62%, 1.18 to 45.96%, and 2.45 to 54.6% for tetanus toxoid Vaccine, HIV/AIDS testing and counseling, danger sign, place of delivery, deworming, iron folic acid, family planning, and breast feeding counseling, from first ANC visit to fourth ANC visit, respectively. Rural residence (AOR = 4.89, 95% CI: 1.21, 19.86), and less than four number of ANC visit (AOR = 5.15, 95% CI: 2.06, 12.86) were significantly associated with inadequate uptake of ANC components.

**Conclusion:**

Only three in hundred pregnant women were received adequate ANC components in the study area. Rural residence and less than four number of ANC visit were factors significantly associated with inadequate ANC uptake. Therefore, the district health department managers and program implementers need to train the health care providers about the components of ANC. As well, increasing community and facility awareness of WHO recommendations on ANC visits focusing on rural women is needed.

## Background

During pregnancy, women and teens receive care from health professionals to ensure the best outcomes for themselves and for their babies [1, 2]. Additionally, it is possible to minimize the risk of maternal death by providing comprehensive maternal health care, throughout their pregnancy, delivery, and afterwards [1, 3].

This is because in Ethiopia the focus ANC is still recommended [4, 5]. The focus of ANC is on maternal care continuum framed as care for the mother, infant, and child based on the following recommended basic packages: The detection and treatment of disorders (such as anemia, abnormal lying, hypertension, diabetes, tuberculosis, syphilis, malaria, and hypertension); the provision of preventive interventions (like tetanus vaccinations and insecticide-treated bed nets); as well as the advice on diet, hygiene, HIV status, birth, emergency preparedness, and baby care and feeding [6, 7].

There have been an estimated 295,000 pregnancies and childbirth related deaths since 2017, with 94% of those deaths occurring in low and lower middle-income countries [8]. An estimated 14,000 maternal deaths occurred in Ethiopia in 2017, contributing to a maternal mortality rate of 401 deaths per 100,000 live births [8]. Increased ANC coverage and quality of healthcare can avert 71% of neonatal mortality, 33% of stillbirths, and 54% of maternal mortality in low and middle-income countries (LMICs) [9]. There is considerable evidence that the effectiveness of ANC is strongly influenced by the essential services covered during visits [10, 11]. Despite improved access to ANC, maternal and neonatal mortality rates continue to rise in developing nations. Negative health outcomes are still prevalent even when coverage is high [12]. Undoubtedly needs an improved ANC services with an attention for the contents of the services [10, 11].

In Ethiopia, ANC visits are at least four times during pregnancy, although eight is the minimum recommended by the World Health Organization (WHO). As part of this visit, every woman should receive all ANC components, including blood pressure measurements, fetal heartbeat, and lying tests, urine tests for infection and protein, syphilis screening, deworming, and nutrition counseling, iron/folic acid supplementation, insecticide-treated bed nets, and tetanus toxoid vaccination [13, 14].

The timing and number of ANC visits were the focus of studies at national and subnational levels rather than the content and associated factors of ANCs [15–19]. Additionally, studies in Ethiopia, were tried to assess components of ANC services, However, almost all of them were not included important packages such as HIV, malaria, as well as the provision of preventive interventions, such as tetanus immunization and insecticide-treated bed nets [20–22]. Therefore, in order to advance maternal health, pregnancy consequences, and child health, this study tried to identify magnitude of inadequate ANC component reception and associated factors in northwest Ethiopia.

## Methods

### Study design, area and period

This is a community based cross sectional survey conducted in Dabat Demographic and Health Survey site (DHSS) from December 10/2020 to January 10/2021. Dabat town is the capital for Dabat district which is about 76 kms far from Gondar town (Zonal town) to the North. According to the Dabat woreda health office report, the projected estimate of the population in the district was 189,944 in 2020/2021. There were a total of 44,789 reproductive women, 25,718 under five children, and 6,401 infants. The Dabat woreda have a total of 36 Kebeles (smallest administrative units in Ethiopia) of which 31 are rural Kebeles. In the district, there are 6 health centers and 29 health posts.

This study is the end line survey of the mega research project on maternal, Neonatal, and Child Health (MNCH) and health services utilization in DHSS sponsored by University of Gondar. The Dabat demographic and health survey (DHS) is one of the six Health and Demographic Surveillance Systems in Ethiopia. The DHSS consists of 13 Kebeles (9 rural and 4 urban).

### Source and study population

The source population was all women in Dabat woreda who gave birth within two years before the survey. The study population was all women in Dabat demographic and Health Survey sites who gave birth within two years before the survey and who were available in their home during the survey. In cases where respondents were not found at home during data collection, interviewers revisited the households a second time, and if they were still unsuccessful, they contacted the next household.

### Exclusion criteria

Women who gave still birth within the last two years and who gave live birth but not alive during the survey were excluded from this study.

### Sample size and sampling technique

The sample size was initially determined using single population proportion formula considering antenatal care coverage, facility delivery, and PNC utilization from the 2016 Ethiopian Demographic and Health Survey report. In the report, the proportion of ANC, facility delivery and PNC utilization in Amhara Region were 67% and 27% respectively and 17% national level PNC utilization within 48 h [23]. Considering the standard normal distribution, the Z-score at 95% confidence level is 1.94; power of 80% and 4%, 3%, and 2% margin of error for ANC and delivery, and PNC services utilization, respectively. The final estimated sample size with a 5% for non-response rate were 558, 884 and 1423 for antenatal care, skilled delivery, and PNC service utilization, respectively. Hence, the largest sample size (1423) was considered as a final sample size. However, the total 2 years extended postpartum women was around to the estimated number, all women who gave birth within two years before the survey were included in this study (i.e. Census was applied). Despite the sample size was calculated using scientific formula, we recruited all pregnant women during the baseline survey and had added more pregnant women using a kind of open cohort and finally had a total 1491 women. Of which, 871 had used ANC which are an appropriate study/source population for this research objective. That is, the rest were not our target population and hence the degree of retention does not affect the result.

#### Variables and measurement

*Dependent variable* to determine the dependent variable, eight components of ANC such as tetanus toxoid Vaccine services, HIV counseling and testing, counseled about danger sign, discussed about place of delivery, deworming services, iron folic acid services, family planning services, and counseling about breast feeding ever in their ANC visit was used. Each component has a binary response (1 = yes and 0 = no). Finally, the dependent variable was categorized as adequate if the women had received all the eight ANC components and codded as “1” and otherwise inadequate and coded as “0”. The construction of the dependent variable was guided by the WHO ANC guidelines and from different literatures [20, 24, 25].

Regarding the independent variables, age was grouped as 16–24, 25–34, and 35–49 years. Residence (urban and rural), current marital status categorized as married and unmarried. No formal education, primary (1–4), midlevel (5–8), high school (9–12), college and diploma, degree and above were categories for education of mothers and their husbands. Of the obstetric characteristics: gravidity (primigravida, multigravida, grand multigravida), parity (primiparous, and multiparous), history of abortion (yes/ no) were included. Health post, health center, hospital and private clinics were categories for place of ANC visit. Number of ANC was grouped as one, two, three and four or more times [20, 24, 25].

### Sampling procedure

The Dabat Demographic and Health Survey sites are determined by University of Gondar, Institute of Public Health from Dabat district representing each agro-ecological zones of the district. This study applied a census method to identify and select eligible study participants.

### Data collection tools and procedures

Questionnaire was developed in English from related literature [7, 26–28] and translated to Amharic, the local language of the area and then translated back to English for consistency and analysis. A structured and pretested questionnaire was used to collect the data. Supervisors and enumerators from the research center were recruited and trained for 5 days on the study objectives, briefed on the content of the questionnaire and procedure prior to fieldwork. Participants' informed consent was obtained and privacy and confidentiality were maintained. Face to face interview technique using a questionnaire was employed to collect the data.

### Data management and analysis

All questionnaires were checked for consistency and completeness. The data were entered in to computer using Epi-data version 3.1 and exported to STATA version 14. Data were cleaned and coded first. Descriptive analysis was done to see the frequency and proportion of dependent and socio-demographic variables. Bivariate and multivariable logistic regression analyses were done to check the presence association between dependent and independent variables.

## Results

### Socio-demographic characteristics of pregnants

A total of 871 pregnant (had at least one ANC visit) women were identified from the survey and included in this study. The mean age of pregnant women was 29.76 ± 6.2 years. About 96.11% were married. Majority (77.15%) of the women were housewife in occupation (Table 1).

**Table 1:** Socio demographic and economic characteristics of pregnant women in Dabat district Ethiopia, DDHS 2020–2021 (n = 871)

Variables	Categories	Frequencies (n)	Percentage (%)
Age (in years)	16–24	165	18.94
	25–34	467	53.62
	35–49	239	27.44
Residence	Urban	444	50.98
	Rural	427	49.02
Current marital status	Married	1433	96.11
	Unmarried	58	3.89
Educational status	No formal education	368	42.25
	Primary (1–4)	95	10.91
	Midlevel (5–8)	130	14.93
	High school (9–12)	157	18.03
	College and diploma	76	8.73
	Degree and above	45	5.17
Occupation	Housewife	672	77.15
	Employed	105	12.06
	Other*	94	10.79
Wealth index	Poorest	126	14.47
	Poorer	176	20.21
	Middle	137	15.73
	Richer	183	21.01
	Richest	249	28.59
Husband education	No formal education	348	39.95
	Primary (1–4)	138	15.84
	Midlevel (5–8)	116	13.32
	High school (9–12)	151	17.34
	College and diploma	48	5.51
	Degree and above	70	8.04
Husband occupation	Farmer	533	61.19
	Employed	180	20.67
	Other**	158	18.14
Family size	3–4	442	50.75
	5–6	331	38.00
	More than 6	98	11.25

*Daily laborer, merchant, private vocational

**Daily laborer, private vocational, merchant

### Obstetric characteristics of pregnant women

The majority (65.21%) of pregnant women were multigravida. Most (96.79%) of pregnant women were attended their ANC at health center (Table 2)

**Table 2:** Obstetric related characteristics of pregnant s in Dabat district Ethiopia, DDHS 2020–2021 (n = 871)

Variables	Categories	Frequencies(n)	Percentage (%)
Gravidity	Primigravida	149	17.11
	Multigravida	568	65.21
	Grand multigravida	154	17.68
Parity	Primiparous	154	17.68
	Multiparous	717	82.32
History of abortion	Yes	20	2.30
	No	851	97.70
Place of ANC	Health post	11	1.26
	Health center	843	96.79
	Other*	17	1.95
Number of ANC	Once	49	5.63
	Twice	172	19.75
	Three times	333	38.23
	Four or more times	317	36.39

*Hospitals, private clinics

### ANC service related characteristics of pregnant

**Fig. 1:**
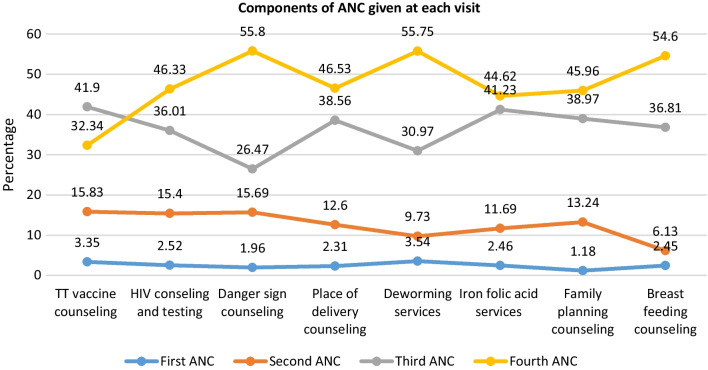
ANC service related characteristics of pregnant women in Dabat district Ethiopia, DDHS 2020–2021 (n = 871) of the pregnant women, 6.77% were not get any of eight (tetanus toxoid vaccine services, HIV counseling and testing, counseled about danger sign, discussed about place of delivery, deworming services, iron folic acid services, family planning services, and counseling about breast feeding ever in their ANC visit) ANC component services. Most (22.50%) of pregnant women were received at least one of the aforementioned ANC components. Overall, 96.67% (95% CI: 95.24, 97.67) had not get adequate ANC components (Table 3)

As shown in Fig. 1 the components of ANC services given to the women are displayed at each ANC visit. In the first ANC visit, 3.35%, 2.52% 1.96%, 2.31%, 3.54%, 2.46%, 1.18%, and 2.45% were received tetanus toxoid Vaccine (TT vaccine), HIV/AIDS, danger sign, place of delivery, deworming, iron folic acid family planning, and breast feeding counselings, respectively. As the number of ANC visit increases, the components of ANC services increased as well: TT vaccine increased from 3.35% to 41.9%, deworming service increased from 3.54% to 55.75% from first ANC visit to fourth ANC visit.

**Table 3:** ANC components related characteristics of pregnant women in Dabat district Ethiopia, DDHS 2020–2021(n = 871)

Sum of ANC components	Frequency (n)	Percentage (95% CI)
0	59	6.77(5.28, 8.65)
1	196	22.50(19.84, 25.40)
2	188	21.58(18.97, 24.45)
3	182	20.89(18.32, 23.73)
4	92	10.58(8.67, 12.79)
5	56	6.42(4.97, 8.27)
6	42	4.82(3.58, 6.46)
7	27	3.09(2.13, 4.48)
8	29	3.32(2.32, 4.75)

### Factors associated with inadequate ANC services

As shown in Table 4, urban residence and number of ANC were significantly associated with the adequacy of received ANC components. Accordingly, the odds of getting inadequate ANC among pregnant women who were from rural residence was 4.89 times higher than urban dweller pregnant women (AOR = 4.89, 95% CI: 1.21, 19.86).

**Table 4:** Bivariable and multivariable regression for inadequate ANC component in Dabat district Ethiopia, DDHS 2020–2021(n = 871)

Variable	Inadequate ANC components		95% CI	
	Yes	No	COR	AOR
*Age*				
15–24	157(95.15)	8(4.85)	1	1
25–34	454(97.22)	13(2.78)	1.77(0.72, 4.37)	1.21(0.38–3.92)
35–49	231(96.65)	8(3.35)	1.47(9.64, 39.96)	1.00(0.22, 4.59)
*Mothers education*				
No formal	360(97.83)	8(2.17)	1.33(0.49, 3.59)	2.77(0.56, 13.69)
Primary	212(94.22)	13(5.78)	0.48(0.19, 1.18)	1.02(0.27, 3.91)
Secondary and higher	270(97.12)	8(2.88)	1	1
*Partner education*				
No formal	338(97.13)	10(2.87)	1.31(0.54, 3.18)	2.04(0.52, 8.01)
Primary	245(96.46)	9(3.54)	1.05(0.42, 2.63)	2.39(0.66, 8.60)
Secondary and higher	259(96.28)	10(3.72)	1	1
*Residence*				
Rural	423(95.27)	21(4.73)	2.6(1.13, 5.94)	**4.89(1.21, 19.86)**
Urban	419(98.13)	8(1.87)	1	1
*Family size*				
3–4	427(96.61)	15(3.39)	1	1
5–6	321(96.98)	10(3.02)	1.12(0.50, 2.54)	1.01(0.24, 4.16)
Above 6	94(95.92)	4(4.08)	0.83(0.27, 2.54)	0.54(0.09, 3.31)
*Wealth index*				
Poor	158(99.37)	1(0.63)	4.51(0.58, 35.1)	7.63(0.84, 69.52)
Middle	264(94.29)	16(5.71)	0.47(0.22, 1.01)	0.75(0.26, 2.13)
Rich	420(97.22)	12(2.78)	1	1
*Birth order*				
Less than 4	509(96.58)	18(3.42)	1	1
4 and more	333(96.80)	11(3.20)	1.07(0.49, 2.29)	0.92(0.20, 4.19)
*History of abortion*				
Yes	18(90.0)	2(10.0)	0.29(0.065, 1.33)	0.34(0.06, 1.98)
No	824(96.83)	27(3.17)	1	1
*Media exposure*				
Yes	352(95.39)	17(4.61)	1.97(0.93, 4.18)	2.28(0.78, 6.67)
No	490(97.61)	12(2.39)	1	1
*Parity*				
Primi para	145(96.67)	5(3.33)	1	1
Multipara	635(96.50)	23(3.50)	0.95(0.36, 2.54)	0.87(0.26, 2.93)
Grand multipara	62(98.41)	1(1.59)	2.14(0.24, 18.64)	3.26(0.25, 42.83)
*Number of ANC*				
1–3	547(98.74)	7(1.26)	5.83(2.46, 13.81)	**5.15(2.06, 12.86)**
4 or more	295(93.06)	22(6.94)	1	1

Bold values indicate *P* value < 0.05

The odds of getting inadequate ANC among women who were attended less than four number of ANC was 5.15 times higher than those who were attended four or more number of ANC visit (AOR = 5.15, 95% CI: 2.06, 12.86).

## Discussion

This study investigated the components of ANC services given during ANC visit. Almost more than nine among ten pregnant women, 96.67% (95% CI: 95.24, 97.67) were not get adequate ANC components which means that only 3.33% of the women were received all components (counselings about TT vaccine, HIV/AIDS, danger sign, iron folic acid, family planning, deworming, breast feeding, and delivery place) of ANC services. This is in line with studies conducted in sub Saharan Africa [29], Nigeria (4.6%) [30]. The finding is lower than studies conducted in Ethiopia ranged from (22.48–27.8%) [20, 31] and in East Africa (11.16%) [21]. The lower prevalence of adequate ANC might be due to the difference in outcome construction in which the previous studies were assessed the ANC components based on six services such as blood pressure measurement, urine tests, birth preparedness advice, nutritional counseling, and information on potential complications. Whereas, the current study considers eight ANC components.

The study identified that rural residence and number of ANC were associated with ANC component uptakes. The finding is supported by studies conducted in Ethiopia [32–34]. The finding of this study reveals that the odds of getting adequate ANC component among pregnant women who were from rural area was lower than urban resident pregnant women. The finding was similar with elsewhere studies conducted in Myanmar, Nigeria, and Bangladesh [32–34]. The possible justification might be due to limited access to transport, rural women may struggle to access healthcare, which can negatively affect their health service utilization [35]. Long distance to the health institutions and less media exposure also accounted for the rural–urban differences [18, 36, 37]. Variations in health seeking behaviors, access to services, and availability of quality healthcare may explain the discrepancy in receiving ANC components across the country. In spite of Ethiopia's continued efforts to improve maternal and newborn health, wide regional disparities remain among the women receiving ANC components [23].

The study identified that the odds of getting inadequate ANC component among women who were attended three or less ANC visit was higher than those who were attended four or more ANC visit. The finding is in congruent with studies conducted in Nigeria [27, 30, 38]. This is possibly due to as the number of ANC visit increases, there will be a high odds of extensive health education sessions for the women from the health care provider that increased the rapport with health care provider, then the women might report the services received as they uptake [39, 40]. It is also proven that frequent contact between ANC provider and the pregnant woman enhances the pregnant woman's familiarity with the health system which enables further uptake of ANC components [41, 42]. This implies that, there is a need to work extensively on pregnant women to have adequate number of ANC visits and obtain ANC packages from skilled health personnel in the health facilities [26].

### Strength and limitations

This study used large sample size. The study might have social desirability and recall bias because the information was self-reported. Since the study is cross sectional, it does not show a temporal relationship between independent variables and the dependent variable. Additionally, due to extensive studies were addressed some components such as blood pressure measurement was omitted it. The study did not include ANC components of abnormal fetal lie, diabetes, tuberculosis, and insecticide-treated bed nets, which would be pertinent if they were incorporated into the construct of ANC components. Which might overestimate the magnitude of inadequate ANC component. Furthermore, qualitative research including women, healthcare providers, and healthcare facilities is also necessary to show the broader picture and identify barriers to receive ANC components.

## Conclusion

Only three in hundred pregnant women were received adequate ANC components in the study area. Being from rural residence and less than four number of ANC visit were factors significantly associated with inadequate ANC uptake. Component wise ANC approach provides evidences for the district health department and program implementers to identify factors directly related with adequate ANC services and to improve the maternal and child health. Therefore, the district health department managers and program implementers need to train the health care providers about the components of ANC. Furthermore, increasing community and facility-level awareness of WHO recommendations on ANC visits focusing rural women also very important.

## Data Availability

The data set is available on a reasonable request from the corresponding author.
